# A cohort study of survival following discharge from hospital in rural Tanzanian children using linked data of admissions with community-based demographic surveillance

**DOI:** 10.1186/s12982-021-00094-4

**Published:** 2021-03-18

**Authors:** Oscar Mukasa, Honorati Masanja, Don DeSavigny, Joanna Schellenberg

**Affiliations:** 1 Ifakara Health Institute (IHI), PO Box 78373, Dar es Salaam, Tanzania; 2 Swiss Tropical and Public Health Institute (Swiss TPH), Socinstrasse, 57, Postfach CH 4002, Basel, Switzerland; 3 University of Basel, Basel, Switzerland; 4 London School of Hygiene and Tropical Medicine, London, United Kingdom

**Keywords:** Demographic, Clinical, Household, Mortality, Children, Cohort, Hospital, Admission, Linked-data, Survival

## Abstract

**Background:**

To illustrate the public health potential of linking individual bedside data with community-based household data in a poor rural setting, we estimated excess pediatric mortality risk after discharge from St Francis Designated District Hospital in Ifakara, Tanzania.

**Methods:**

Linked data from demographic and clinical surveillance were used to describe post-discharge mortality and survival probability in children aged < 5 years, by age group and cause of admission. Cox regression models were developed to identify risk factors.

**Results:**

Between March 2003 and March 2007, demographic surveillance included 28,910 children aged 0 to 5 years and among them 831 (3%) were admitted at least once to the district hospital. From all the children under the demographic surveillance 57,880 person years and 1381 deaths were observed in 24 months of follow up. Survivors of hospital discharge aged 0–5 years were almost two times more likely to die than children of the same age in the community who had not been admitted (*RR* = 1.9, *P* < 0.01, 95% CI 1.6, 2.4). Amongst children who had been admitted, mortality rate within a year was highest in infants (93 per 1000 person years) and amongst those admitted due to pneumonia and diarrhoea (97 and 85 per 1000 person years respectively). Those who lived 75 km or further from the district hospital, amongst children who were admitted and survived discharge from hospital, had a three times greater chance of dying within one year compared to those living within 25 km (adjusted HR 3.23, 95% CI 1.54,6.75). The probability of surviving the first 30 days post hospitalization was 94.4% [95% CI 94.4, 94.9], compared to 98.8% [95% CI 97.199.5] in non-hospitalized children of the same age in the commuity.

**Conclusion:**

This study illustrates the potential of linking health related data from facility and household levels. Our results suggest that families may need additional support post hospitalization.

## Background

Infectious diseases pose a serious threat to child health in developing countries. Malaria, pneumonia and diarrhoea make up a major part of the morbidity and mortality burden among children aged less than 5 years in resource limited settings [1–4]. Yet in circumstances where effective curative care is available through hospital services, fatal outcome in the weeks and months after discharge from hospital has been reported in a relatively high number of young children who survived the acute phase of their illness, suggesting that such children remain vulnerable for an extended period of time beyond their initial recovery. Inspite of the available literature regarding factors for survival of young children in rural settings following discharge from hospital [5], there is in resource limited settings, public health importance in optimizing evidence regarding potential of linking data of admissions with community-based demographic surveillance. On the Kenyan coast, more than seven fold higher mortality among children discharged from hospital than amongst similarly-aged children in the community was reported, along with 4.5% (14,971 children) cumulative mortality risk within one year of discharge [6]. Apart from disease specific factors as was reported in other areas [7, 8] excess mortality post hospitalization could be due in part to social factors. For example in one study in Guinea-Bissau self-discharge was noted as the main risk factor after adjusting for other determinants of childhood mortality [9]. In this study there was 2.5 times higher mortality risk during the first 2 weeks amongst children aged less than 5 years who were discharged from hospital, compared to mortality levels in the community.

Here we report the one year survival of children post hospitalization, based on individual bedside data linked with community-based demographic surveillance records using cohort of 28,910 children aged less than 5 years who were followed for an average of 48 months. We also investigated risk factors for survival post-hospitalization including proximity from the hospital to the home village and household relative wealth status.

## Methods

### Study area and source of data

**Fig. 1:**
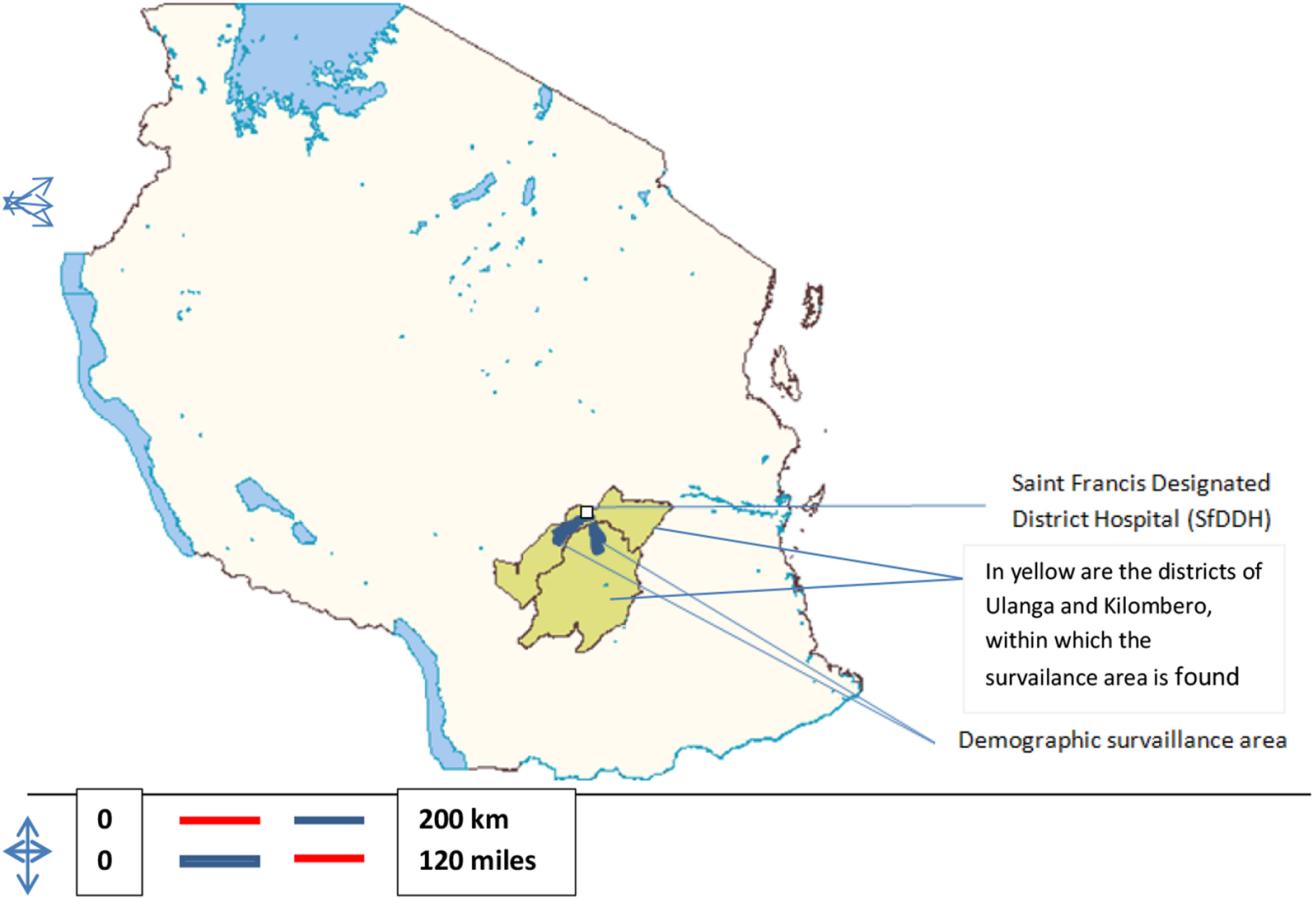
Map of Tanzania showing two districts of the Ifakara HDSS and location the SFDDH Ifakara HDSS SFDDH, Saint Francis District Designated Hospital; HDSS, Household and Demographic and Surveillance System

The data came from linked demographic and clinical surveillance systems within Ifakara demographic surveillance area in Kilombero and Ulanga districts, in south-eastern Tanzania. The Health and Demographic Surveillance System (HDSS) generated household based data on residence, health status and vital events [10–12]whereas the Clinical Surveillance System (CSS) which was run at the St. Francis Designated District Hospital (SFDDH) in Ifakara town documented admission data for children aged 15 years and less [13]. The population under HDSS depended to a large extent on SFDDH for clinical services. Figure 1 shows a map of Tanzania showing the districts of Kilombero and Ulanga and location of the SFDDH.

### Health system structure in Tanzania and the characteristics in the study area

There is hierarchically a public health care system in Tanzania that is made of dispensaries, Health Centers (HCS), District Hospitals (DH), Regional Hospitals (RH) and tertiary Hopstals (TH) at zonal and national levels. In line with that, national treatment guidelines specify what health services should be offered at which facility type, and in which cases treatment should be referred to a higher facility level. Dispensaries are the health posts at the grassroot and first point of contact with the health system. Most common illnesses are therefore treated at this level and somewhat at Health Centers before they are refered up wards in case of relatively more severe cases. Any conditions thus, which require in-patient care are referred from the dispensaries to next level, the Health Centers (HCs) and then further up. Formal distinction between dispensaries and Health Centers (HC) is that while the former exclusively provide out-patient care, the later should be able to provide around-the-clock care to patients. Hospitals at the districts and further higher up have extra capacity along with much more sofisticated services as the levels goes up.

The study area, HDSS, covered 25 villages (13 in Kilombero and 12 in Ulanga) districts and constituted more than 124,000 people (Majige, majige). There were six and eight health facilities in Kilombero and Ulanga HDSS areas respectively [14]. Saint Francis Designated District Hospital (SFDDH) is the district hospital for Kilombero, serves a population of 331,167 and is located at Ifakara town. The population in town, Ifakara, which were not part of the HDSS, was 45,700 during the study period, according to the national census of 2002 [14].

Kilombero had only two non-governmental hospitals both located around 75 km apart in the northern part of the district. SFDDH has a capacity of 372 beds and provides in patient services to people not only from Kilombero district but also to those from the neighboring districts, Ulanga, Kilosa and Morogoro rural [15]. For Kilombero and Ulanga districts as a whole, beyond HDSS area, there was one district hospital in Ulanga, seven [7] HCs and 41 dispensaries all together [16].

### Health and demographic surveillance system

This was started in 1996 to collect data longitudinally on demographics, health and survival at household level. Between January 1997 and March 2007 data on residence and vital events including pregnancies, births, deaths, and migrations (in and out) have been updated by a team of trained interviewers who visit every household every 4 months [10]. In December 2007 approximately 96,000 residents were registered in the HDSS, living in approximately 19,000 households. Other data included educational level, possession of household assets for generation of an index of relative socioeconomic status, and distance (in km) of the center of the child’s village to the SFDDH.

### Clinical surveillance system

This involved round-the-clock registration of inpatient paediatric illnesses on admission to SFDDH. Particulars of the child were recorded on arrival by a clinical officer trained to administer surveillance procedures. They also recorded history of the presenting illness along with symptoms on admission. For every child a blood slide was taken for malaria and read according to standardized quality-controlled laboratory procedures, which are further described elsewhere [17]. The clinical officers took physical measurements and observed the child, made a diagnosis, decided on treatment and documented an outcome of the admission. Detailed list of the variables recorded within HDSS and CSS are summarized in annex 1.

### Linkage of HDSS data with admission records (CSS)

Paediatric admissions at SFDDH came from within and outside the demographic surveillance area. For individuals who live within the area, HDSS records can potentially be linked to admission data. In the Tanzanian health system mothers or guardians bring the child’s Reproductive and Child Health (RCH) card whenever they seek curative services. Based on availability of their RCH cards during encounters at the district hospital we developed a mechanism to link individual HDSS and CSS records. Linking was facilitated by a unique HDSS ID printed on a sticker which was stuck onto every child’s RCH card during one of the 4-monthly HDSS home visits. For children whose HDSS based IDs were not available during hospitalization, admission records remain unlinked with HDSS data. Not all children had HDSS based IDs available on RCH cards at the time of hospital encounter, for reasons including that the child was not yet a registered member of the HDSS (s/he was either born or migrated in after the most recent HDSS home visit), and that mothers/guardians did not bring RCH cards with them when they came for curative services at the hospital. We therefore searched the HDSS database for all such children, and HDSS interviews made additional home visits to verify the identity of children whose ID was not captured during a hospital encounter.

### Inclusion and exclusion criteria

Children were included in the study if they were registered in the HDSS and aged less than 5 years at any point in time during the period March 2003 to March 2007, referred to as the “entry period”. They entered the study at birth, by migration into the study area or by having been resident in the demographic surveillance area at start of the entry period, whichever came last. They subsequently contributed time into the study until they migrated out of the area, reached the age of 5 years, died, or the end of follow up, whichever came first. The HDSS model allows for tracking of time at risk for children who moved house within the demographic surveillance area. A set of HDSS rules and procedures, further described elsewhere, guided processes to register births and deaths and account for migration within and outside surveillance area [10]. For the current study we considered two cohorts of children (1) all children less than 5 years who were registered members of the HDSS and were not admitted–the “general cohort” and (2) those from the HDSS who were admitted and who survived their hospital admission. In order to be included in the non-admission cohort, children had to be "unexposed" for at least 12 months before start of the study timeframe, which is equivalent time to the period under which the exposed ones where followed for an event of interest and likewise an admission was included only if it happened during timeframe of the study.

### Statistical methods

Stata 12.0 was used for all analyses. Time points of the “beginning”, “mid” and “end” of the entry period were defined at 15th March 2003, 15th March 2005 and 15th March 2007 respectively. We selected, for computation of wealth quintiles, household wealth variables which were common at all the three time points of the entry period. At each of these points we summarized household relative socioeconomic status- (SES) from principal components analysis [18–20] as well as number of children for each cohort by district (whether on the North or South side of the main river which divides Kilombero and Ulanga districts in the demographic surveillance area), age, sex and proximity of child’s home village to the district hospital. Four equal intervals of distance (proximity) to hospital, < 25 Kms, 25 to < 50 Kms, 50–75 Kms, 75 + Kms were considered in the analysis. Main as well as underlying causes of admission were also summarized for the sick children and discharge survivals.

Mortality rates were computed for the general cohort, overall and by age as well as estimated mortality Rate Ratio (RR) by age, between admitted and non admitted groups of children. For the “sick cohort” mortality rates were computed by causes of admission. Statistical significance was assessed using χ^2^ test and rate ratio for comparison of groups, and survival functions were estimated using Mantel–Haenszel methods. Cox regression analysis was used to estimate, amongst survivors of hospital discharge, the association between mortality risk and covariates including Body Mass Index (BMI) on admission, proximity of home village to district hospital, household wealth status, whether or not the child lived with biological father, mother or both, as well as the main cause of admission. Univariate analysis was used to ascertain the factors for inclusion in the full model [P < 0.05].

Confounding by age was adjusted for through splitting follow–up time. Two way interactions were tested to determine if the effect of one of the variables differed depending on the level of the other variable.

## Results

**Fig. 2:**
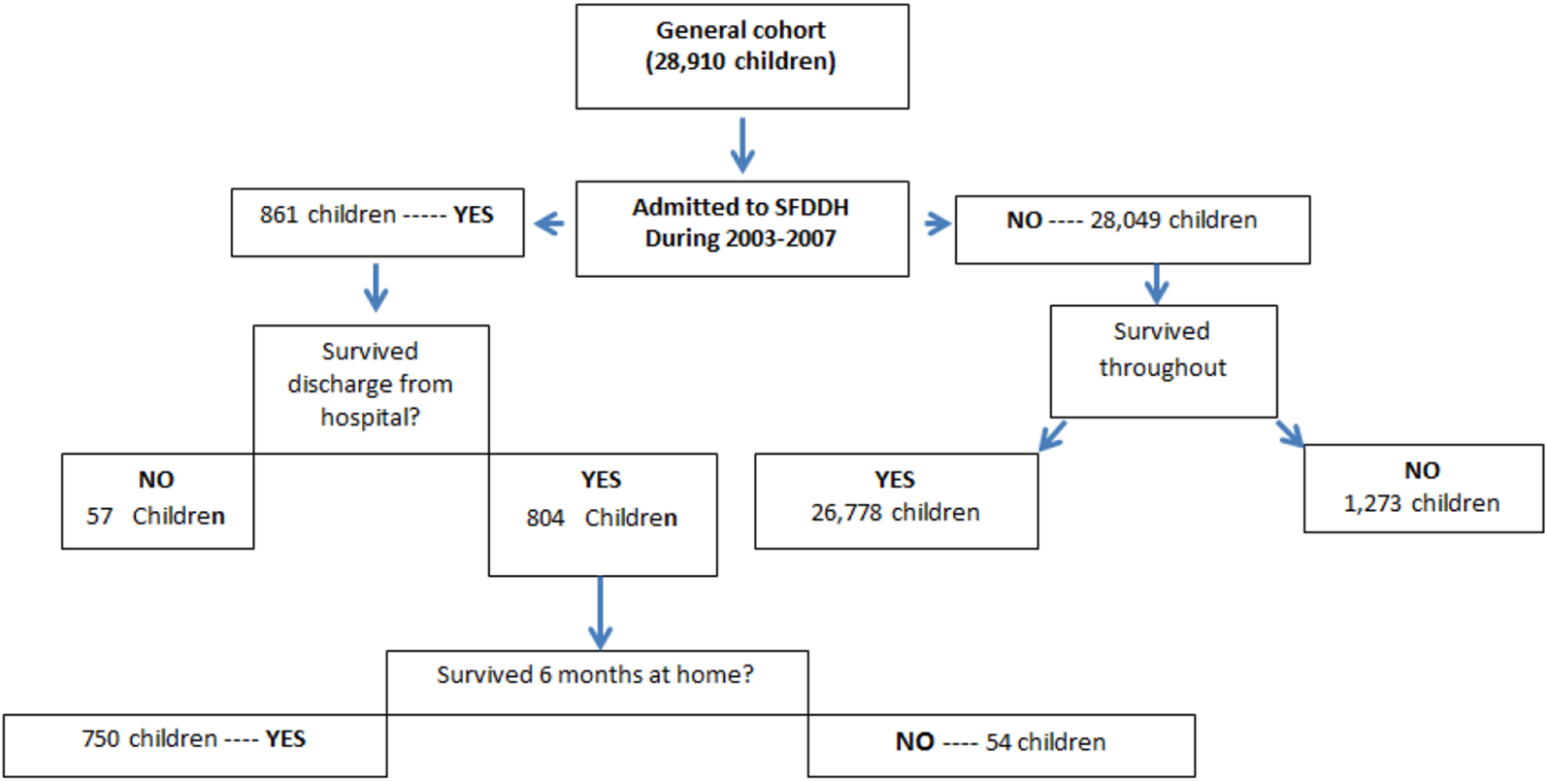
Flow chart showing number of children and deaths in the cohort study survival analysis

The number of children and deaths in the cohort study survival analysis are shown in Fig. 2. Out of 1,147 children who were claimed to be from the the HDSS cohort, who were admitted at the district hospital during the period of follow up, 861 (75%) had their HDSS and hospital admission records successfully linked. Failure to link the remaining quarter could be due to incorrect or sometimes false addresses given by mothers or guardians when they presented at the district hospital. In the latter case those from outside service area of the district hospital sought privileges which were offered to children from within the service area. Another reason was that the mothers or guardians and hence the child had not qualified for HDSS membership yet at the time of admission. There was no evidence that linking varied by sex, age, proximity of their home villages from the district hospital and side of the HDSS area they came from (data not shown). There were 57 deaths among children from the HDSS area who were admitted to hospital and had linked records (case fatality rate [CFR] = 7.0%) and 750 (93%) of hospital discharge survivors survived at least a further 6 months period.

Table 1 shows a mid-study snapshot of demographic and socioeconomic characteristics of children who formed the general, “sick and post-discharge survival” cohorts of the HDSS from 2003–2007. Wealth status amongst the HDSS households were heterogeneously distributed across wealth categories and likewise number of children across one year age bands from infants to 4–5 years old. Average age was 30 months in the general cohort, and 27 months for both the sick and post discharge survival cohorts. Slightly more than half of the children, in all the three cohorts, lived in Kilombero, rather than Ulanga district (58%) and 60% lived more than 25 km away from the district hospital.

**Table 1:** Distribution of demographic and socioeconomic variables amongst HDSS children during 2003–2007, during 2005 as mid-point of entry period

	General cohort, N = 28,910	Sick cohort, N = 703	Discharge survivals, N = 661
Age in months	n (%)	n (%)	n (%)
Infants	3,119 (19)	166 (24)	158 (24)
1–< 2	3,418 (21)	161 (23)	150 (23)
2–< 3	3,143 (20)	166 (23)	152 (23)
3–< 4	3,049 (19)	127 (18)	120 (18)
4–< 5	3,446 (21)	83 (12)	81 (12)
Mean	30	27	27
Sex: Male	8,076 (50	351 (50)	326 (49)
Female	8,099 (50)	352 (50)	335 (51)
District:			
Kilombero	9,466 (59)	389 (55)	367 (56)
Ulanga	6,709 (42)	314 (45)	294 (44)
Proximity^*b*^			
< 25 km	2,657 (16)	284 (40)	267 (40)
25–< 50 km	5,095 (32)	213 (30)	202 (31)
50–< 75 km	3,940 (24)	124 (18)	116 (18)
75 + km	4,483 (28)	82 (12)	76 (11)
*Wealth quintiles*^*a*^			
Poorest	3,397 (21)		
Very poor	3,235 (20)		
Poor	3,397 (21)		
Less poor	2,912 (18)		
Least poor	3,235 (20)		

^a^The following household wealth variables were considered all across from beginning to end of entry period for computation of household relative socio economic (wealth) status: *Main occupation of the head of household; ownership of bicycle, radio, poultry, bed net, house made from blocks, house has latrine, type of water source and time to water source: *Showing number of children in each of the 5 quintiles

^*b*^Proximity (in Kms) from home village to district hospital;

Mortality rates per 1000 person years are shown in Table 2. There were 57,880 person years contributed by 28,910 childrens in the HDSS among whom 1384 died during the follow up. Admitted children had double the mortality rate of the non-admitted, 49 (95% CI 40.6, 59.2) and 23 (95% CI 21.6, 24.2) respectively. In Table 3 are mortality Rate Ratios (RR) by age between admitted and non-admitted groups of children in the general cohort of HDSS children. In all age groups mortality rates for admitted children were higher than those for non-admitted children, with the difference being most marked for children aged 3–4 years (RR 3.7 (2.5, 6.1))”.

**Table 2:** Mortality rate /1000 person-years in HDSS cohort of children during 2003–2007 (n = 28,910)

By admission status	Time at risk person years	No. of deaths	Rate (CI)
Admitted	2,202.9	111	49.0 (40.6, 59.2)
Not admitted	55,677.8	1,273	22.9 (21.6, 24.2)
By age in years			
< 1	11,106.0	800	72.0 (67.2, 77.2)
1–< 2	12,199.5	283	23.2 (20.6, 26.1)
2–< 3	11,847.7	146	12.2 (10.4, 14.4)
3–< 4	11,629.2	77	6.5 (5.2, 8.2)
4–< 5	11,098.3	78	6.9 (5.5, 8.7)
By broad age categories			
1–4	46,774.7	581	12.5 (11.5, 13.5)
0–4	57,880.0	1,384	23.9 (22.6, 25.2)

CI: Confidence Interval; broad age categories

**Table 3:** Mortality Rate Ratio (RR) between admitted and non-admitted children, by age in the HDSS cohort

Age in years	RR (95% CI)	χ^2^	*P* value
< 1	1.3		
1–< 2	2.7 (0.9, 1.8)		
2–< 3	3.9 (1.9, 3.9)		
3–< 4	3.7 (2.5, 6.1)		
4–< 5	1.5 (1.9, 7.4)		
1–4	3.3 (0.5, 4.9)		
0–4^đ^	2.1 (2.5, 4.2)		
Overall estimate controlling for age	1.9 (1.8, 2.6)	49.4	< 0.01
Approximate test for unequal RRs (effect modification)	21.5 (1.6, 2.4)		< 0.01

CI: Confidence Interval; broad age categories

Results of univariate Cox regression analysis for mortality risk factors in the post-discharge survival cohort are summarized in Table 4. Children who lived in a village 75 km or more from the district hospital had a higher chance of dying compared to those living within 25 Kms (HR 3.23, 95% CI 1.54,6.75), adjusted for other risk factors which were included in the study.

**Table 4:** Cox regression analysis of risk factors for mortality in the post discharge survival cohort, n = 804

	Hazard ratio	P value	95% CI
Lived with both biological parents	1.55	0.11	0.92, 2.65
Lived with biological father	1.57	0.10	0.91, 2.69
Lived with biological mother	1.06	0.87	0.53, 2.11
Was related to head of the household	1.10	0.77	0.57, 2.15
Improper BMI on admission date	1.21	0.52	0.68, 2.17
Socioeconomic status			
Least Poor households^a^	1.00	–	–
Less poor households	0.55	0.20	0.22, 1.37
Poor households	1.04	0.93	0.48, 2.24
Very poor households	1.13	0.76	0.52, 2.44
Poorest households	0.88	0.79	0.34, 2.29
Cause of admission			
Malaria^a^	1.00	–	–
Anemia	0.89	0.82	0.37, 2.22
Pneumonia	1.20	0.74	0.41, 3.43
Diarrhoea	0.57	0.39	0.16, 2.09
Others	0.65	0.24	0.31, 1.34
Proximity			
< 25 km^a^	1.00	–	–
25– < 50 km	0.92	0.84	0.43, 2.01
50– < 75 km	1.48	0.32	0.68, 3.23
75 + km	3.55	< 0.01	1.77, 7.11

Household wealth status is computed at midpoint of entry period, 2005

CI: Confidence interval; BMI: Body Mass Index

^a^Reference for hazard ratio calculation

**Fig. 3:**
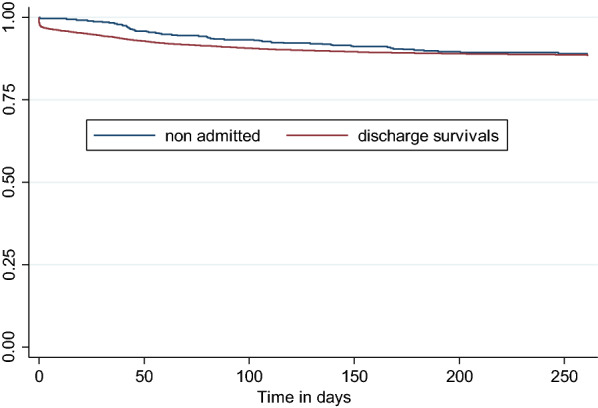
Kaplan–Meier estimates of hospital survivals and Non-hospitalized children in Ifakara HDSS during 2003–2007. SFDDH, Saint Francis Designated District Hospital; HDSS, Household and Demographic Surveillance System

In Fig. 3 are Kaplan–Meier survival estimates of survivors of hospital discharge and non-hospitalized children in Ifakara HDSS during 2003–2007. In the first 30 days following hospital discharge, the survival rate was 94.4% [95% CI 94.4, 94.9] compared to 98.8% [95% CI 97.1,99.5] in non-hospitalized children of the same age. Survival probability post-discharge remained lower than in non-hospitalised children for the following 6 months, with convergence at 7 months 89.4% [95% CI 86.3,91.8] versus. 88.9% [95% CI 88.389.5]. Mortality rates associated with pneumonia and diarrhoea were much higher (97 and 85 per 1000 person years respectively) than for malaria and anaemia (33 and 49 per 1000 person years respectively) which were other two leading main causes of hospital admission. Pneumonia as an underlying cause of hospital admission had a higher mortality rate (88 per 1000 person years) compared to other causes and the mortality rate associated with malaria was higher as an underlying than main cause of admission (64 versus 33 per 1000 person years).

## Discussion

### Research potential of linked data

Linking administrative records data with survey data helps to overcome the shortcomings of administrative data discussed above by enhancing the usually limited set of information recorded. Not only does this linkage increase the number of variables, but the information that is already routinely collected for administrative purposes can be complemented by additional survey information to capture fundamental economic concepts in a way that is more amenable to economic research.

Demographic and clinical data systems originally operated independently from each other, yet together they offered an opportunity to generate linked data on illness and child survival in a defined study population living in a poor rural area. The value-added as a result of linking the data has been demonstrated. For example, through the discovery that mortality rates in admitted children was double that of non-hospitalized children and also this study shows that among admitted kids, the rate mortality rate in-hospital and for 6 m post discharge is double and hence suggesting for further attention on post discharge management of children. Without linked data, such an insight could have not been possible. The HDSS and CSS services in their original states have been described elsewhere [21, 22] including facilitation of understanding of community based factors for morbidity, mortality and utilization of health services as well as measurement of performance in clinical services, patient health outcomes at bedside and evaluation of clinical interventions respectively.

Overall case fatality rate (7%) was high, but fairly consistent with what was recorded before in the same area [17] and elsewhere in studies of hospital admissions [5, 23]. The possible causes of the high number of deaths in-hospital in this study include large number of admissions due to pneumonia and diarrhoea which are amongst the most difficult childhood conditions in resource limited settings. Excess mortality among hospital discharge survivors as compared to those who had not been admitted have been documented in other studies of similar nature, including an association with admission due to gastroenteritis, malaria and acute respiratory tract infections [7], abnormal Blantyre Coma Scale score as well as HIV-positive status [8]. Adding to this evidence, our study showed a relationship between the proximity of a child`s home from the district hospital and post-discharge survival, with three fold higher chance of dying amongst children who lived at least 3 times away compared to those at immediate locality of the district hospital. This kind of an investigation could have not been possible in the original unlinked state of the HDSS and CSS, and builds on previous work using an independent CSS in the same area, which reported that living at least 10 kms away from the district hospital was related to risk of being admitted with anaemia [24].

### Limitations of the study

Linkage requires a unique identifier (ID) for each individual. In this case there was time lag for some of the children in obtaining such IDs due to survailance criteria before one can be a registreted member. As such linkage become burdensome and matching quality suffered.

## Conclusion

Synergy across health related data sources can produce public health benefits greater than the sum of the individual parts, particularly in settings of limited resources.

## Data Availability

The datasets are not publicly available but are available from IHI via corresponding author on request.

## Annex 1

See Table 5

**Table 5:** Type of data collected from Demographic and Clinical Surveillance Systems

Category of the Variable	Demographic surveillance system
General	Age, sex
Baseline	Village, sub village, household affiliation, name, date of birth, mother or father (biological) living in the same household
Routine	Quarterly (four monthly) updates on residence and demographic events (birth,death,migration-in and out, marital status change,
Category of the Variable	Clinical Surveillance System^a^
General	Age, sex, date of birth, admission date, date of discharge from hospital
Symptoms	Fever, breast feeding normally, cough, history of difficulty in breathing, fits, diarrhoea, type of stools, vomiting, dysentery, hematuria
Signs	Auxiliary temperature, pulse, pallor, chest in-drawing, nasal flaring, deep breathing, crackles\crepitations, neck stiffness, bulging fontanelle, spleen size, liver size, dehydration, sunken fontanelle, oral candida, wheeze/rhonchi, impaired consciousness, able to recognize pain, abnormal positioning, flaky paint skin, orange hair, jaundice
Physical measurements	Height, weight and mid upper circumference (MUAC)
Laboratory	Parasitemia, blood glucose concentration, packed cell volume (PCV)
Diagnosis and treatment	Diagnosis (main and underlying cause of admission),treatment and outcome of the admission

*Clinical surveillance system*

^*a*^*:Laboratory methods: Thick blood films were air-dried and stained with Giemsa before being read twice, independently, by separate* slide readers. The slide reading results were compared by a computer program which generated a list of slides with conflicting results, then read a third time by a senior slide reader. PCVs were read using a Hawkesley hematocrit reader at IHRDC after centrifugation of capillary blood in micro-capillary tubes

## Abbreviations

HDSS: Household demographic surveillance system
CSS: Clinical surveillance system
SFDDH: St. Francis Designated District Hospital
RCH: Reproductive and child health
SES: Socioeconomic status
RR: Ratio
BMI: Body mass index
CFR: Case fatality rate
